# Evidence for increased olfactory receptor gene repertoire size in two nocturnal bird species with well-developed olfactory ability

**DOI:** 10.1186/1471-2148-9-117

**Published:** 2009-05-25

**Authors:** Silke S Steiger, Andrew E Fidler, Bart Kempenaers

**Affiliations:** 1 Department of Behavioural Ecology and Evolutionary Genetics, Max-Planck Institute for Ornithology, Eberhard-Gwinner Strasse, 82319 Seewiesen, Germany; 2 Cawthron Institute, 98 Halifax Street East, Private Bag 2, Nelson 7042, New Zealand

## Abstract

**Background:**

In vertebrates, the molecular basis of the sense of smell is encoded by members of a large gene family, namely olfactory receptor (OR) genes. Both the total number of OR genes and the proportion of intact OR genes in a genome may indicate the importance of the sense of smell for an animal. There is behavioral, physiological, and anatomical evidence that some bird species, in particular nocturnal birds, have a well developed sense of smell. Therefore, we hypothesized that nocturnal birds with good olfactory abilities have evolved (i) more OR genes and (ii) more intact OR genes than closely related and presumably less 'olfaction-dependent' day-active avian taxa.

**Results:**

We used both non-radioactive Southern hybridization and PCR with degenerate primers to investigate whether two nocturnal bird species that are known to rely on olfactory cues, the brown kiwi (*Apteryx australis*) and the kakapo (*Strigops habroptilus*), have evolved a larger OR gene repertoire than their day-active, closest living relatives (for kiwi the emu *Dromaius novaehollandiae*, rhea *Rhea americana*, and ostrich *Struthio camelus *and for kakapo the kaka *Nestor meridionalis *and kea *Nestor notabilis*). We show that the nocturnal birds did not have a significantly higher proportion of intact OR genes. However, the estimated total number of OR genes was larger in the two nocturnal birds than in their relatives.

**Conclusion:**

Our results suggest that ecological niche adaptations such as daily activity patterns may have shaped avian OR gene repertoires.

## Background

In vertebrates, the detection of odorous chemicals in both air and water, is mediated by olfactory receptors (ORs)[1]. ORs are members of the superfamily of seven transmembrane G-protein coupled receptors (GPCRs) and are expressed in olfactory neurons of the olfactory epithelium [[1], for review, see [2]]. OR gene coding regions are short (~1 kb) and intronless [1,2]. Both the total number and the number of intact (i.e. putatively functional) OR genes vary greatly amongst the genomes of vertebrate taxa [3]. For example, there are about 150 OR genes in the zebrafish genome [4], 280 – 550 in the chicken genome [5-7] and, typically, >1000 OR genes in mammalian genomes [[7], for reviews, see [8,9]]. Amongst primates, the proportion of intact OR genes is significantly reduced in humans (~50%) when compared with other apes (~70%) [[10,11], but see [12]], a finding hypothesized to reflect decreasing behavioral reliance on the sense of smell during human evolution. More generally, it has been suggested that the numbers, and proportions, of intact OR genes in a vertebrate genome correlates with olfactory acuity at the behavioral level [3].

For birds, visual and auditory cues play important roles in behaviors as diverse as foraging, mate attraction and territory defense [13,14]. However, the behavioral significance of avian chemosensation is still debated. Increasing evidence, from both behavioral and morphological studies, suggests that olfactory ability in at least some bird species, in particular nocturnal birds, is excellent and may even be equivalent to the olfactory abilities of mammals [[15], for reviews, see [16-18]].

Concordantly with these studies the OR gene repertoire in the red jungle fowl (*Gallus gallus*) genome, as estimated from analysis of the draft *G. gallus *genomic sequence, is surprisingly large, in the range of 220 – 550 OR paralogues [5-7]. More recent evidence suggests that both the intact proportion (73 – 96%) and the estimated total number (100 – 660 copies) of OR genes in the genomes of nine distantly related avian species are much higher than expected when working from the assumption that birds have a poorly developed sense of smell [19]. Interestingly, the total number but not the proportion of intact OR genes was correlated with olfactory bulb ratio (OBR)[19], a possible morphological correlate of olfactory ability [20].

The aim of this study is to investigate the OR gene repertoires of two sets of closely related species, where one species in each group is nocturnal and known to rely on olfactory cues. This work builds on a previous study, where we examined the OR gene repertoires of distantly related species [19]. The two nocturnal bird species are the brown kiwi (*Apteryx australis*) and the kakapo (*Strigops habroptilus*). Both night-active species are flightless and do not have well-developed night vision or hearing [[21], R.J. Moorhouse, pers. comm.], but do have a well-developed sense of smell and relatively large olfactory bulbs [21-23]. Furthermore, the brown kiwi and kakapo belong to entirely different evolutionary lineages, evolved their nocturnal behavior independently and have extant diurnal relatives which facilitate comparative studies (brown kiwi relatives: emu *Dromaius novaehollandiae*, rhea *Rhea americana*, ostrich *Struthio camelus*; kakapo relatives: kaka *Nestor meridionalis*, kea *Nestor notabilis*). We hypothesized that both nocturnal birds had evolved more OR genes and more intact OR genes than their day-active relatives.

As genome sequences are not available for any avian species other than the red jungle fowl (*Gallus gallus*), we used two complementary methods to estimate OR gene repertoire size. Southern hybridisation was used to provide a relative measure of the number of OR gene sequences in the avian genomes. While having the advantage of directly hybridising to the genomic DNA, Southern hybridisation does not provide information about whether the hybridising sequences are intact genes or pseudogenes. Therefore, the PCR, using degenerate primers directed at the OR gene family, and subsequent sequencing were used to estimate both the percentage of intact and the total number of OR genes in the avian genomes. Both methods have been used previously to estimate OR gene repertoire sizes in fish [24,25], amphibians [26], birds [27,28] and mammals [29,30]. The OR gene repertoire estimates obtained using these two methods were compared and interpreted in the light of the evolutionary histories and ecological adaptations of the brown kiwi and kakapo.

## Methods

### Blood samples collection

Blood samples for this study were kindly provided by Gyula Gajdon (University of Vienna, Austria; kea), Manuel García Hartmann (Duisburg zoo, Germany; elegant-crested tinamou), Ron Moorhouse (Department of Conservation, New Zealand; kaka and kakapo), Gregor Müller (Ulm zoo, Germany; rhea) and André Schüle (Berlin zoo, Germany; brown kiwi). Blood collection procedures conformed to the animal experimental ethics regulations of the German Federal Republic, the European Union and New Zealand. International transport of DNA samples conformed to the legal requirements of the Convention on the International Trade of Endangered Species (CITES).

### PCR amplification of partial olfactory receptor (OR) genes

Blood samples were stored in Queen's lysis buffer before genomic DNA was isolated using a commercial kit (DNeasy tissue kit; Qiagen, Hilden, Germany). The design of PCR primers to amplify avian OR partial coding sequences has been described in detail in [19]. Briefly, degenerate primers were designed to anneal to coding sequences corresponding to evolutionarily conserved sequence motifs within transmembrane (TM) regions 3 and TM 7 of ORs. As a subset of the avian ORs, termed γ-c, is greatly expanded in number within avian genomes, two different sets of PCR primers were required; those targeting mainly the more diverse non-γ-c OR genes and those mainly targeting the more homogenous γ-c OR genes. To amplify non-γ-c OR sequences three different forward primers were used in combination with three different reverse primers. Forward primers: 5'-ATG GCI TAY GAY MGI TA-3' [31], 5'-GCI ATG GCI TAY GAY MGI TA-3' [32] and 5'-ATG GCI TAY GAY MGI TAY STI GCI ATY TG-3' [27]; reverse primers: 5'-TA DAT IAG IGG RTT IAG CAT IGG-3' [19], 5'-AR ISW RTA DAT RAA IGG RTT-3' [32] and 5'-GG RTT IAR CAT IGG-3' [31]. Amplifications were conducted using each forward primer in combination with each reverse primer thereby generating nine different PCR products. The conserved sequence motif within the TM3 region of the γ-c ORs differs significantly from that of the non-γ-c ORs so alternative forward primers were required for amplifying γ-c OR partial coding sequences [19]: forward primers: 5'-ATC TGY AAR CCI YTI CAY TA-3' and 5'-RTT GCI ATY TGY AAR CCY CTR CAC TA-3' which correspond to the γ-c OR TM3 motifs ICKPLHY and VAICKPLHY, respectively. The two γ-c OR forward primers were used in combination with the reverse primer 5'-AR ISW RTA DAT RAA IGG RTT-3' [32] thereby generating PCR products consisting largely of γ-c OR clade sequences. All primer pairs were predicted to generate products of approximately 0.5 kb which represents approximately half the full OR coding sequence. The PCR reaction conditions were: 2.0 mM Mg^2+^, dNTPs (0.1 mM); primers (0.8 μM); 1 U *Taq *DNA polymerase (MBI Fermentas, St. Leon Rot, Germany) and genomic DNA (100 ng) template in a final volume of 50.0 μl with thermocycling parameters: 94°C/2 minutes, 1 cycle; 94°C/30 seconds; 37°C/30 seconds, ramping from 37°C to 72°C at 0.2°C/second; 72°C, 60 seconds; 5 cycles; 94°C/30 seconds; 45°C/30 seconds; 72°C, 60 seconds; 30 cycles; 72°C/7 minutes; 4°C/hold. Amplification products were separated through 2% (w/v) agarose gels (Nusieve GTG agarose, BioWhittaker Molecular Applications, Rockland, U.S.A.) and products of ~0.5 kb cloned and sequenced as described in [19]. Note that amplification products generated using the non-γ-c and the γ-c OR clade primers were pooled using equal volume aliquots before the ligation reaction. Therefore there were two ligations: one using the nine non-γ-c amplicons and one using the two γ-c amplicons.

### Sequence editing and analysis

Electropherograms were visually inspected and low-quality sequences discarded. PCR primer sequences were deleted and sequences sharing ≥ 98.5% identity, determined using the Sequence Identity Matrix function of BioEdit [33], were considered to be amplified from a single OR orthologue [19]. This procedure was used to accommodate errors that may have been introduced during the amplification process. To confirm that the sequences were partial OR coding sequences, each was used as a query string in BLASTX searches of the GenBank non-redundant (nr) database. Sequences were shifted into the correct reading frame using a custom-written PERL program. For each species, 50 individual clones were analyzed (25 clones derived by using each of primer pairs that generally amplified the non-γ-c and the γ-c OR clade partial coding sequences).

### Estimation of the proportion of intact OR genes and the total number of OR genes

We designated a sequence as intact if an uninterrupted coding region was found (i.e. sequence without stop codon), and as a pseudogene when an interrupted coding region was found (i.e. sequence containing a stop codon; Gilad et al. 2004). Note that this method may overestimate the proportion of intact OR genes, because frame-shift mutations located outside of the amplified partial coding region (~TM3 – TM7) or in promoter regions will not be detected [10,11]. In two cases, apparent amplicons derived from the same gene were designated both intact and pseudogene and these were excluded for the calculation of the proportion of intact OR genes but not for the estimation of the total number of OR genes (see below). Chi-square tests were calculated to compare the proportion of intact OR genes between the nocturnal birds and their diurnal relatives using SPSS 15.0 (SPSS, Chicago, IL, USA).

As described previously in Steiger et al. (2008) [19], a nonparametric estimation technique applying the concept of 'sample coverage" [34] was used to estimate the total number of OR genes in each genome investigated. This method estimates the total number of OR genes from an incomplete sampling of OR genes (achieved via PCR). In a first step, the number of times identical PCR products were re-sequenced was used to estimate sample coverage (C) and its coefficient of variation (CV). In a second step, we chose the appropriate coverage estimator (ACE1) given the information provided by C and CV. This method does not assume an equal probability for each gene to be cloned and thus accounts for primer bias. Abundance coverage estimators, their standard errors, confidence intervals and related statistics for all species were calculated using the software SPADE [35] and can be found in Additional file 1. In a previous study, we estimated the jungle fowl OR gene repertoire to consist of 638 genes [19], which was close to the previous estimate of 550 [7]. This suggested that our method provides a sufficiently reliable estimate of OR gene repertoire sizes in species for which full genomic sequences are not yet available.

We did not estimate the functional OR repertoire size because the two estimated variables required to calculate it (i.e. the total number of OR genes and the proportion of those that are intact) already have substantial measurement errors, so that their multiplication would lead to an even more uncertain estimate. Instead, we use the total number of OR genes as a proxy for the number of functional OR genes. This is meaningful, because in vertebrates the total number of OR genes correlates positively with the total number of intact OR genes (see Additional file 2).

To show that the estimates for the kakapo and the kiwi were significantly higher than those of their relatives, 1-tailed t-tests were conducted (psittaciform comparison: kakapo vs. kea, kakapo vs. kaka; paleognath comparison: brown kiwi vs. emu, brown kiwi vs. ostrich, brown kiwi vs. rhea). Subsequently, Fisher's method [36] was used to obtain a single p-value for each comparison.

### Southern Hybridization

Genomic DNA (10 μg) was digested with each of four restriction endonucleases (*Eco*RI, *Pst*I, *Hind*III and *Taq*I; Fermentas, St. Leon-Rot, Germany) and the digestion products separated via electrophoresis through 0.8% agarose gels before transfer to positively-charged nylon membranes (Roche, Germany; Whatman Schleicher & Schuell, Dassel, Germany) using a vacuum blotting system (VacuGeneXL; Amersham Biosciences, Freiburg, Germany) following the manufacturers instructions. Ethidium bromide staining was used to confirm that equivalent amounts of total digested genome DNA were loaded in each lane.

**Figure 1:**
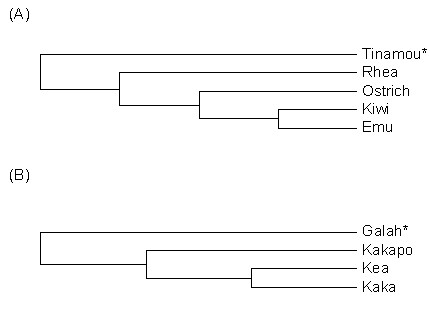
**Phylogenetic relationships among avian taxa compared in this study**. (A) Ratite phylogenetic tree topology adapted from reference [37]. Note that a recent study places the tinamou within the ratites [39]. (B) Partial pssitaciform phylogenetic tree, topology derived from reference [38]. Outgroup taxa used for the generation of OR gene probes for Southern hybridizations are indicated by an asterisk. Branch lengths were arbitrarily set to 1.

For a comparative Southern hybridization study, such as this, is critical that the labeled probes used for the hybridizations are derived from an 'outgroup' taxon that, while related, is evolutionarily equidistant from all the genomes being compared. Therefore, based on the phylogenetic tree topologies [37,38] shown schematically in Figure 1, OR probes were generated from the elegant-crested tinamou (*Eudromia elegans*; fam. Tinamiformes, outgroup to the ratites) and the galah (*Elophus roseicapillus*; fam. Cacatuidae, outgroup to the Nestoridae), respectively. Note that a recent study places the tinamou within the ratites [39] and that kakapo have also been classified as the sole member of a distinct family Strigopidae [40]. For probe generation, partial OR coding sequences were amplified using the degenerated primers targeting the non-γ-c OR genes (see above) and cloned into pGemT-easy. Plasmids were digested with *Eco*RI (Fermentas, St. Leon-Rot, Germany) and the inserts isolated from agarose gels (QIAquick Gel Extraction kit, Qiagen, Hilden, Germany) and labeled with digoxigenin (DIG) using a commercial kit (DIG high prime DNA Labeling and Detection Starter Kit I, Roche, Germany).

OR partial coding sequences amplified from the tinamou and galah were aligned with 78 known, putatively functional *G. gallus *OR receptor genes. [from ref [7], sequences listed in Supporting data set 8, ] using ClustalW [41]. For the alignment, *G. gallus *nucleotide sequences between transmembrane domain (TM) 3 and TM7 were used. We used the Neighbor-Joining (NJ) method and Poisson-distances to construct phylogenetic trees using the MEGA software package (version 4.0; [42,43]). The reliability of the phylogeny was evaluated with 1000 bootstrap repeats.

**Figure 2:**
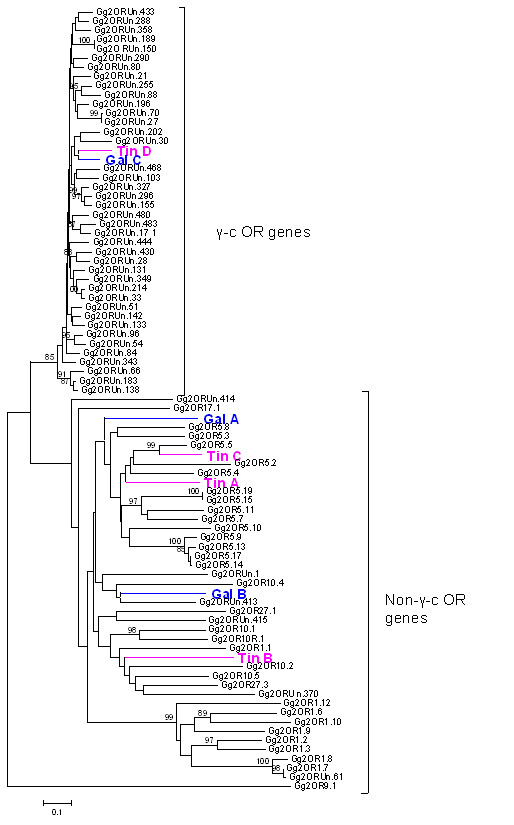
**Neighbor-joining (NJ) phylogenetic tree**. Neighbor-joining (NJ) phylogenetic tree generated from an alignment of all predicted red jungle fowl (*Gallus gallus*) putatively functional OR nucleotide sequences (n = 78) as described in reference [7]. Positions of seven sequences corresponding to the partial avian OR cDNA sequences used as probes in the Southern blotting probes are indicated: TinA-D and GalA-C. Probes Gal-C and Tin-D are clearly placed within the γ-c OR clade while probes Gal-A, B and Tin-A, B, C are placed within the non-γ-c OR clade. Bootstrap values > 80% are indicated. The scale-bar indicates the number of nucleic acid substitutions per site. *Abbreviations*: Tin, elegant-crested tinamou, Gal, galah; Gg, *Gallus gallus*; OR, olfactory receptor.

Four tinamou and three galah OR partial coding sequences were selected from divergent regions of the OR gene family for use as Southern hybridization probes. The selected probes were denoted, along with their corresponding GenBank accession numbers, Tin-A [GenBank: EU599489], Tin-B [GenBank: EU599490], Tin-C [GenBank: EU599491], Tin-D [GenBank: EU599492] and Gal-A [GenBank: EU599486], Gal-B [GenBank: EU599487] and Gal-C [GenBank: EU599488] (Figure 2). Identities of the probe nucleotide sequences ranged between 49 – 66% and therefore were considered lower than the threshold of cross-hybridization under the hybridisation conditions used [44] (Additional file 3).

Blots were hybridized with ~25 ng/ml probe overnight at 37°C and/or 42°C. Note that due to the limited amount of genomic DNA available from the kakapo we conducted Southern blots comparing the OR gene repertoire of the kakapo, kea and kaka only at 42°C hybridization temperatures and thus, under more conservative conditions.

Washes (2 × 5 min in 2 × SSC, 0.1% SDS at 15–25 °C; 2 × 15 min in 0.5 × SSC, 0.1% SDS at 65°C) and labeled probe visualization with nitroblue tetrazolium (NBT)/5-bromo-4-chloro-3-indolyl phosphate (BCIP) followed the manufacturers protocol. The membranes were photographed using a digital gel documentation system (Gel Doc 2000, BioRad, Hercules, California, USA). Brightness and contrast of the images were optimized using Adobe Photoshop (version 9.0, Adobe Systems, San Jose, California, USA).

Note that Southern hybridizations were run in 'duplicates' with the *Eco*RI/*Hind*III and *Taq*I/*Pst*I restrictions run together on the same gels. Thus the *Eco*RI/*Hind*III and *Taq*I/*Pst*I results arise from different gels/blots/hybridizations. Therefore, it is valid to compare the results for different species within a given enzyme/probe combination.

## Results

### Estimation of the total number of OR genes

The total numbers of distinct OR sequences amplified using degenerate PCR primers, with the seven avian genomes as templates varied between 22 and 42 (Table 1) [GenBank: EU594675 – EU594890; EU599486 – EU599492, for more details, see Additional file 4]. The mean number of distinct OR sequences was similar in both clades (mean ± SEM: Paleognathae: 32 ± 4, Psittaciformes: 30 ± 5).

**Table 1:** Overview of the species studied and their OR gene repertoires.

Common name	Scientific name	Order	Family	Activity pattern	No. distinct OR sequences amplified^c^	No. pseudogenes amplified^c^	% intact OR genes	Total no. OR genes	Confidence interval (lower/upper)^d^
***Paleognath comparison***									
Brown kiwi	*Apteryx australis*	Apterygiformes	Apterygidae	N	42 (27/15)	9 (5/4)	78.6	478	(156/1708)
Emu	*Dromaius novaehollandiae*	Casuariiformes	Dromaiidae	D	31 (25/6)	6 (6/0)	80.6	109	(56/275)
Greater Rhea	*Rhea americana*	Rheiformes	Rheidae	D	28 (11/17)	7 (4/3)	75.0	66	(40/156)
Ostrich	*Struthio camelus*	Struthioniformes	Struthionidae	D	25 (21/4)	4 (2/2)	84	58	(34/143)
Elegant crested tinamou^a^	*Eudromia elegans*	Tinamiformes	Tinamidae	D	N.A.	N.A.	N.A.	N.A.	N.A.
									
***Psittaciform comparison***									
Kakapo	*Strigops habroptilus*	Psittaciformes	Nestoridae^b^	N	38 (14/24)	12 (0/12)	68.4	312	(122/932)
Kaka	*Nestor meridionalis*	Psittaciformes	Nestoridae	D	22 (12/10)	5 (1/4)	77.3	55	(31/155)
Kea	*Nestor notabilis*	Psittaciformes	Nestoridae	D/C	30 (17/13)	2 (0/2)	93.3	102	(52/263)
Galah^a^	*Elophus roseicapillus*	Psittaciformes	Cacatuidae	D	N.A.	N.A.	N.A.	N.A.	N.A.

^a ^used as outgroup

^b ^Kakapo have also been classified as the sole member of a distinct family Strigopidae [40]

^c ^Numbers in brackets refer to non-γ-c and γ-c OR genes, respectively

^d ^95% confidence interval of the estimate (lower/upper)

Activity pattern, number of distinct OR sequences and pseudogenes amplified, predicted proportion of potentially intact OR genes, estimated number of OR genes and lower and upper confidence interval of the estimate for seven bird species. Note that the number of distinct OR sequences is the sum of the number of intact OR sequences and the number of distinct OR pseudogenes. Abbreviations: N = nocturnal, D = diurnal, C = crepuscular, N.A. = not applicable.

The estimated total number of OR genes in each genome varied between 55 and 478 (Table 1). The estimated OR gene repertoires of the nocturnal species (kakapo and kiwi) were 5 to 8 times larger than those found in their diurnal closest relatives (Table 1). The estimate for the brown kiwi was significantly higher than that of its relatives (Fisher's combined p = 0.047). Similarly, there was a strong trend for the kakapo to have a larger OR gene repertoire than its relatives (Fisher's combined p = 0.060).

### Estimation of the proportions of potentially intact OR genes

Among the paleognath species the proportions of intact OR genes did not differ significantly (mean ± SEM: 79.6% ± 1.9%; χ^2 ^= 0.7, df = 3, P = 0.87). Although the mean proportion of intact OR genes was similar in the three psittaciform genomes (79.9% ± 7.5%), the proportions differed significantly between the three species (χ^2 ^= 6.3, df = 2, P = 0.04). Notably, the proportion of intact OR genes was significantly higher in the kea and kaka genomes than in the kakapo (Table 1). The lower estimated percentage of intact OR genes in the kakapo genome reflects the large number of pseudogenes (12) amplified from the kakapo γ-c OR clade (Table 1).

### Comparison of OR gene repertoire sizes using Southern blot hybridization

#### A) Southern hybridizations of palaeognath genomes

**Figure 3:**
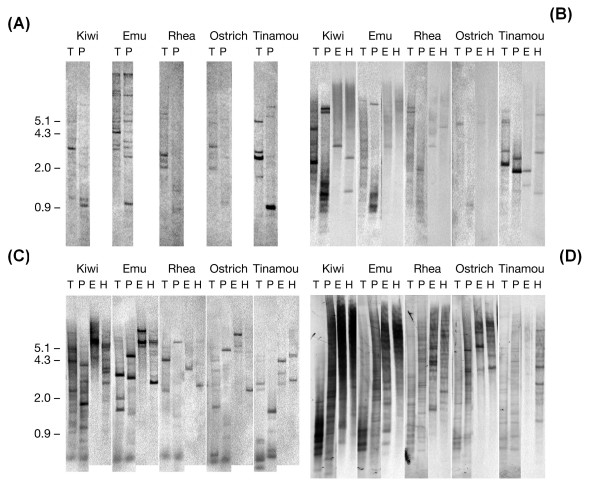
**Southern hybridization of restriction enzyme digested palaeognath genomic DNAs**. Genomic DNAs isolated from five palaeognath taxa (ostrich, emu, brown kiwi, greater rhea, elegant-crested tinamou) were digested with four different restriction enzymes and used for Southern hybridization with four DIG-labeled probes generated from elegant-crested tinamou partial OR coding sequences: (A) probe Tin-A [GenBank: EU599489], (B) probe Tin-B [GenBank: EU599490], (C) probe Tin-C [GenBank: EU599491] and (D) probe Tin-D [GenBank: EU599492]. 37°C hybridization conditions were used for the results shown. Abbreviations: T, *Taq*I; P, *Pst*I; E, *Eco*RI; H, *Hind*III; DIG, digoxigenin; Tin, elegant-crested tinamou. Approximate positions of the size standards (kb) are indicated. Note that each figure was generated by splicing together images from two separate blots (see Methods for details).

Genomic DNA samples isolated from brown kiwi, emu, greater rhea, ostrich and elegant-crested tinamou (positive control) blood samples were digested with four different restriction enzymes and transferred to a filter before hybridization (37°C), with the OR probes, Tin-A, B, C and D (Figure 3). Quantitative comparisons of the number of bands labeled in different species, and different hybridizations, proved problematic. Therefore the Southern hybridization results were interpreted qualitatively.

The Tin-A probe labeled the most bands in the emu DNA with broadly similar numbers of bands detected in the kiwi, ostrich and rhea DNA (Figure 3A). As expected, the fragments of tinamou DNA were most strongly labeled (Figure 3A).

The Tin-B probe labeled, with low intensity, few rhea and ostrich DNA fragments (Figure 3B). In contrast, the tinamou, emu and kiwi DNA had a number of strongly labeled bands. In particular the *Taq*I and *Pst*I digested kiwi DNA revealed a number of strongly labeled fragments (Figure 3B). The Tin-C probe labeled multiple bands in all four genomes. However, labeling of the kiwi and emu DNA fragments appears the most intense with an indication of there being more labeled fragments in the kiwi DNA (Figure 3C). It is noteworthy that the Tin-A and Tin-C probes generated entirely different banding patterns despite being placed quite close together on the OR phylogenetic tree and sharing 66% sequence identity (Figure 2). The Tin-D probe, which encodes a γ-c OR partial sequence, generated more bands than the other three Tin probes when hybridized with the DNA of all four species (Figure 3D). Indeed both the kiwi and emu DNA samples appear to have so many hybridizing fragments that the result is a smear (Figure 3D). Overall the kiwi DNA appears much more intensively labeled than that of the other species with emu DNA the second most intensively labeled. Somewhat surprisingly, given that the probes used were derived from tinamou, the tinamou DNA is the least intensely labeled of the four species (Figure 3D).

#### B) Southern hybridizations of psittaciform genomes

Genomic DNA isolated from kakapo, kea, kaka and galah blood was digested with four different restriction enzymes and transferred to a filter before hybridization at 42°C with three galah OR gene probes, Gal-A, B and C (Figure 2).

**Figure 4:**
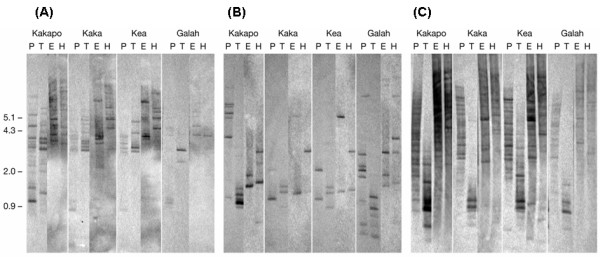
**Southern hybridization of restriction enzyme digested psittacciform genomic DNAs**. Genomic DNAs isolated from four psittaciform taxa (kaka, kea, kakapo and galah) were digested with four different restriction enzymes and used for Southern hybridization with three DIG-labeled probes generated from galah partial OR coding sequences: (A) probe Gal-A [GenBank: EU599486], (B) probe Gal-B [GenBank: EU599487] and (C) probe Gal-C [GenBank: EU599488]. 42°C hybridization temperatures were used for all results shown. Abbreviations: T, *Taq*I; P, *Pst*I; E, *Eco*RI; H, *Hind*III; DIG, digoxigenin, Gal, galah. Approximate positions of the size standards (kb) are indicated. Note that each figure was generated by splicing together images from two separate blots (see Methods for details).

In all four pssitaciform taxa, multiple fragments of varying intensity were labeled with the Gal-A and Gal-B probes (Figure 4A, B). The fragment labeling patterns for the kea and the kaka are strikingly similar for both probes with all four restriction enzymes reflecting the close evolutionary relationship of these congeneric species (Figure 4A, B). In contrast, the fragment patterns of the kakapo, are quite distinct from those of the kea and kaka, particularly in the *Taq*I and *Pst*I digests (Figure 4A, B). Overall the kakapo lanes appear to contain more bands or, in the case of the TaqI/Gal-B combination, a much more intense band than the kea and kaka or, indeed, the positive control galah (Figure 4A, B).

The Gal-C probe labeled the largest number of bands when hybridized to all four pssitaciform genomes (Figure 4C), which is consistent with its placement within the large γ-c clade (Figure 2). It would appear that the intensity of the 'smear' in the kakapo lanes is stronger than in the other taxa samples (Figure 4C). This difference is perhaps most prominent in the TaqI digested samples where the intensity of the hybridization to the kakapo DNA is clearly stronger than to the kea, kaka and even the galah.

The observed inter-taxa differences in hybridization intensities cannot be attributed to differences in the amounts of DNA loaded on the gels as because ethidium bromide staining of the gels revealed little differences in DNA quantities/labeling intensities between lanes (data not shown).

## Discussion

The majority of OR partial coding sequences amplified in this study did not contain stop codons, suggesting that they were derived from potentially functional OR genes. Indeed the degree of interspecific variation in the proportion of intact genes appears to be small. This is consistent with previous work, which showed a high (~80%) proportion of intact OR genes in nine bird genomes from seven different orders, including the brown kiwi and the kakapo [19]. The estimate of the proportion of intact genes in the kakapo genome was higher in our previous study (i.e. 82% versus 68% in this study), possibly due to the slightly different method used (the PCR products were pooled in this study and thus there were two ligation reactions, whereas previously they were not pooled and there were twelve ligation reactions). Notably, the estimates obtained from the two different procedures are not significantly different (χ^2 ^= 2.3, df = 1, P = 0.12).

If the proportion of intact OR genes is an indicator of olfactory ability, one would expect nocturnal birds with good olfactory abilities to encode a higher proportion of intact OR genes than related, but diurnal species. Contrary to this hypothesis, we found that (i) the estimated proportion of intact OR genes were lower for the nocturnal kakapo than for the kea and the kaka and (ii) did not differ between the brown kiwi and its diurnal relatives. However, the estimated total number of OR genes was 5 to 8 times larger in the two nocturnal species than in their diurnal relatives, even though our estimate of the kakapo OR gene repertoire was smaller than in the previous study [19]. The observed differences in OR gene repertoire sizes are remarkable, but perhaps not surprising for the following two reasons. First, birds also show remarkable interspecific variation in the size of the olfactory bulb (OBR) (relative to brain size). The OBR is the ratio of the greatest diameter of the olfactory bulb relative to the greatest diameter of the cerebral hemisphere, expressed as a percentage [22]. For example, the OBR in the Snow petrel, *Pagodroma nivea*, is twelve times larger (37%) than in the Black-capped chickadee, *Poecile atricapillus *(3%) [22]. Thus, similar interspecific variation in OR gene repertoire size can be expected. Second, in mammals, OR gene repertoire sizes range from 606 OR genes in the macaque to 2129 OR genes in the cow [3]. Thus, OR gene repertoire sizes also vary greatly among mammalian species.

In agreement with our previous study [19], our data suggest a positive correlation between the relative size of the olfactory bulb and the number of OR genes. It appears that it is the total number of OR genes, rather than the proportion of intact OR genes, that is most closely linked with olfactory dependency in the bird species examined here. Our results therefore support the notion that the proportion of intact OR genes "is not always a good criterion for the study of the evolution of OR genes" [45]. Interestingly, recent evidence suggests that OR pseudogenes are expressed in the olfactory epithelium in humans, suggesting that pseudogenes may have a biological function [46]. These results imply that future studies should investigate both intact OR genes and pseudogenes more carefully [45].

The results of the Southern blot analyses, while only qualitative, generally agree with the estimates of OR gene numbers based on PCR. Both nocturnal species showed generally more or stronger bands than their diurnal closest relatives. In general, variable band intensity is presumed to reflect varying levels of sequence homology between the "target" and probe sequences. However, the fact that the intensity of hybridization to the kakapo and brown kiwi DNA is much stronger than that to the galah and the tinamou (i.e. the species from which the probes were derived) suggests that multiple kakapo and brown kiwi sequences hybridized with the probes. This was particularly clear in the expanded γ-c OR gene clade, indicating that at least in this clade the two nocturnal species have a greater total number of OR genes.

In this study, two molecular techniques, the PCR and Southern hybridization, were used to compare the numbers of OR genes, both intact and non-functional, encoded in seven avian genomes. Both techniques have their limitations. Firstly, the PCR, using degenerate primers, may overestimate the number of intact OR genes, as these may have stronger conservation of primer annealing sites than do non-functional pseudogenes [10]. Secondly, only half of the OR open reading frame (ORF) was amplified and sequenced and therefore, mutations occurring in the remaining N- and C-termed coding regions would not be detected. Finally, due to unpredictable primer bias, some OR genes may amplify preferentially relative to others. Thus, the ratios of OR partial coding sequences amongst the amplification products may not represent a random sample of the OR repertoires in the template DNA [10,11]. The limitation of the Southern blot method is that it only estimates the number of OR genes in a given subfamily and does not provide information about the functionality of these genes.

Olfactory receptor genes evolve dynamically via duplication and/or gene conversion in a process that has been called 'birth and death evolution' [3,47]. Why would it be advantageous for a nocturnal bird to have evolved more, and perhaps more diverse, olfactory receptor genes? It has been suggested that the more OR genes encoded in an organism's genome the finer the animal's discrimination amongst odor molecules is [4]. Thus, differences in the size of the OR gene repertoire may cause different odor sensitivities among birds. A wide receptor repertoire is also likely to allow binding/detection of many, structurally-diverse, 'odorous' compounds [48]. Thus, a nocturnal bird that has evolved more OR genes might be able to smell more diverse odorants than a diurnal bird that lives in the same habitat. Thus higher numbers of OR genes may contribute to the ability of nocturnal species to locate food at night via olfactory cues [23,49]. Before the relatively recent colonization of New Zealand by humans, this archipelago lacked terrestrial mammalian predators [50]. It is therefore perhaps not surprising that these nocturnal birds have adapted to the ecological niche usually occupied by mammals elsewhere. This includes the development of such mammalian-like characteristic as reliance upon olfactory information [21]. It should be noted that the ability to detect odorants most likely does not only rely on the number of OR genes, but also on differences in olfactory epithelium size and in the number of olfactory neurons [8]. Unfortunately, very little information exists about these traits in birds [for review, see [16]].

In addition to a role in foraging, birds also use olfactory abilities in a variety of other contexts such as navigation [51,52], predator detection [53], nest-building [54,55] and conspecific and mate recognition [56]. Furthermore, birds seem to lack both the vomeronasal organ and vomeronasal receptors, which are thought to mediate social chemo-communication in mammals [5]. Thus, it remains to be shown whether and how birds detect pheromones. The recent observation that pheromones can be detected by both the vomeronasal and olfactory epithelia in mammals is striking [57], because this implies that avian pheromones, if they exist, could potentially act via the conventional sense of smell (i.e. olfactory epithelia and ORs). Alternatively, trace amine-associated receptors (TAARs), a new class of "olfactory" receptors could be relevant for avian social communication and individual recognition [58,59].

Interestingly, the olfactory anatomy is also well developed in procellariiform seabirds (petrels, albatrosses and shearwaters) and olfactory cues such as krill-related odors or odors associated with phytoplankton play an important role in foraging behavior [22,60]. So far, procellariiform OR genes have received little attention [19]. Therefore, future studies could investigate whether the reliance on olfactory cues in seabirds is reflected in their OR gene repertoires. For example, it would be interesting to determine whether burrowing petrels that return to their nest at night have evolved a larger OR gene repertoire than surface-nesting petrels that rather rely on visual cues to recognize their nest [61].

Besides enhancement of the olfactory system, some nocturnal birds have compensated for the reduced effectiveness of vision at night by an enhanced sense of vision (e.g. owls (Koenig, Becking, 1999), or by increasing other capacities such as hearing (owls, (Koenig, Becking, 1999)) or echolocation (oilbirds and swiftlets) (Konishi, Knudsen, 1979; Medway, 1959). Further work could also address whether nocturnal birds that invested in enhanced visual or auditory perception have smaller olfactory receptor repertoires, which would suggest a trade-off between investments in vision versus olfaction.

## Conclusion

In summary, our data indicate that the OR gene repertoires are larger in the genomes of two nocturnal bird species than in their most closely related, but diurnal, species. Such results confirm and extend previous behavioral studies suggesting that some nocturnal bird species have a well-developed sense of smell. Our results strongly suggest that ecological niche adaptations such as daily activity patterns, and associated sensory changes, may have shaped, or been shaped by, avian OR gene repertoires. In general our results support the growing consensus that birds – in common with mammals – may rely on their sense of smell in a variety of important life-history contexts.

## Abbreviations

BCIP: 5-bromo-4-chloro-3-indolyl phosphate; DIG: Digoxigenin; Gal: Galah; GPCR: G-protein coupled receptor; NBT: Nitroblue tetrazolium; NJ: Neighbor-Joining; OBR: olfactory bulb ratio; OR: olfactory receptor; PIC: phylogenetically independent contrasts; Tin: elegant-crested tinamou; TM: transmembrane domain.

## Authors' contributions

Each author contributed towards the conception and design of the experiments. SSS conducted the experiments, analyzed the data and wrote the manuscript. AEF and BK helped writing and contributed to the critical revision of the manuscript. All authors read and approved the final manuscript.

## Supplementary Material

Additional file 1 **Abundance coverage estimators**. Abundance coverage estimators and related statistics for seven avian species. The abundance coverage estimator ACE_1 was used, calculated as described in additional reference [3]. Click here for file

Additional file 2 **Correlation between the number of intact OR genes and the total number of OR genes in vertebrates**. Positive correlation between the number of intact OR genes and the total number of OR genes in vertebrates (r = 0.95, p < 0.001, n = 13). Numbers were obtained from reference [45]. Click here for file

Additional file 3 **Sequence identities between the probes**. Sequence identities (in %) between (A) galah and (B) elegant-crested tinamou probes on the nucleic acid level. Click here for file

Additional file 4 **Sequence information**. Summary of (a) paleognath and (b) psittaciform partial olfactory receptor (OR) sequences generated in this study. Copies indicate how often a partial OR sequenced was amplified. Click here for file
