# Supplementary material for: Evidence for increased olfactory receptor gene repertoire size in two nocturnal bird species with well-developed olfactory ability
Source: BMC Evol Biol. 2009 May 25;9:117. doi: 10.1186/1471-2148-9-117 (PMC2701422; doi:10.1186/1471-2148-9-117)
Supplement: Additional file 1 — Abundance coverage estimators. Abundance coverage estimators and related statistics for seven avian species. The abundance coverage estimator ACE_1 was used, calculated as described in additional reference [3]. [file 1471-2148-9-117-S1.doc]

| Species | Estimator^a^ | Estimate | CI95_low^b^ | CI95_high^c^ | Est_se^d^ | n^e^ | D^f^ | C^g^ | CV^h^ |
| --- | --- | --- | --- | --- | --- | --- | --- | --- | --- |
| ***Paleognath comparison*** | | |  |  |  |  |  |  |  |
| Brown kiwi | ACE_1 | 477.8 | 156.0 | 1707.4 | 336.6 | 50 | 42 | 0.260 | 0.920 |
| Emu | ACE_1 | 108.6 | 56.2 | 274.6 | 49.2 | 50 | 32 | 0.540 | 0.740 |
| Ostrich | ACE_1 | 58.2 | 34.4 | 142.6 | 23.9 | 50 | 25 | 0.700 | 0.738 |
| Rhea | ACE_1 | 66.4 | 39.5 | 156.2 | 26.0 | 50 | 28 | 0.660 | 0.699 |
| ***Psittaciform comparison*** | | |  |  |  |  |  |  |  |
| Kaka | ACE_1 | 55.4 | 30.9 | 154.9 | 26.5 | 50 | 23 | 0.790 | 0.975 |
| Kakapo | ACE_1 | 312.3 | 122.1 | 932.0 | 181.6 | 50 | 38 | 0.360 | 0.946 |
| Kea | ACE_1 | 102.1 | 52.4 | 262.6 | 47.2 | 50 | 30 | 0.580 | 0.804 |

**Additional file 1**

^a^Abundance coverage estimator for heterogeneous samples

^b^Confidence interval of the estimate (low boundary)

^c^Confidence interval of the estimate (high boundary)

^d^Standard error of the estimate

^e^Number of plasmids sequenced

^f^Number of distinct OR partial coding sequences

^g^Estimation of the sample coverage

^h^Estimation of the coefficient of variation
