# Supplementary figures and images for: Evidence for increased olfactory receptor gene repertoire size in two nocturnal bird species with well-developed olfactory ability
Source: BMC Evol Biol. 2009 May 25;9:117. doi: 10.1186/1471-2148-9-117 (PMC2701422; doi:10.1186/1471-2148-9-117)

**Additional file 2**

**
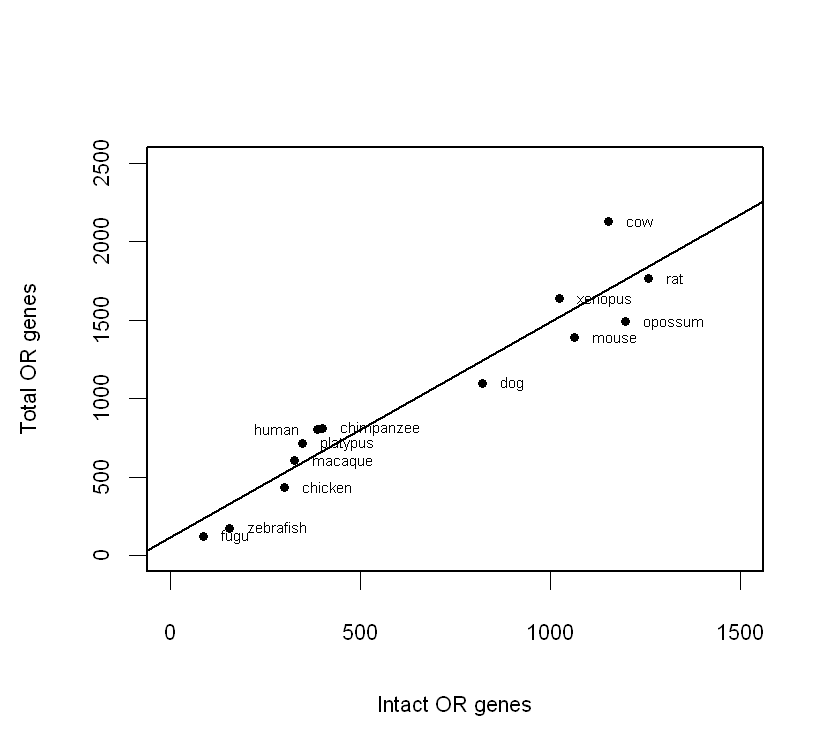
**

Supplement: Additional file 2 — Correlation between the number of intact OR genes and the total number of OR genes in vertebrates. Positive correlation between the number of intact OR genes and the total number of OR genes in vertebrates (r = 0.95, p < 0.001, n = 13). Numbers were obtained from reference [45]. [file 1471-2148-9-117-S2.doc]
