# Supplementary material for: Evidence for increased olfactory receptor gene repertoire size in two nocturnal bird species with well-developed olfactory ability
Source: BMC Evol Biol. 2009 May 25;9:117. doi: 10.1186/1471-2148-9-117 (PMC2701422; doi:10.1186/1471-2148-9-117)
Supplement: Additional file 3 — Sequence identities between the probes. Sequence identities (in %) between (A) galah and (B) elegant-crested tinamou probes on the nucleic acid level. [file 1471-2148-9-117-S3.doc]

**Additional file 3**

(A)

| **PROBES** | **Gal_A** | **Gal_B** | **Gal_C** |
| --- | --- | --- | --- |
| **Gal_A** |  | 56 | 51 |
| **Gal_B** | 56 |  | 49 |
| **Gal_C** | 51 | 49 |  |

(B)

| **PROBES** | **Tin_A** | **Tin_B** | **Tin_C** | **Tin_D** |
| --- | --- | --- | --- | --- |
| **Tin_A** |  | 52 | 66 | 52 |
| **Tin_B** | 52 |  | 56 | 49 |
| **Tin_C** | 66 | 56 |  | 56 |
| **Tin_D** | 52 | 49 | 56 |  |
