# Supplementary material for: Evidence for increased olfactory receptor gene repertoire size in two nocturnal bird species with well-developed olfactory ability
Source: BMC Evol Biol. 2009 May 25;9:117. doi: 10.1186/1471-2148-9-117 (PMC2701422; doi:10.1186/1471-2148-9-117)
Supplement: Additional file 4 — Sequence information. Summary of (a) paleognath and (b) psittaciform partial olfactory receptor (OR) sequences generated in this study. Copies indicate how often a partial OR sequenced was amplified. [file 1471-2148-9-117-S4.doc]

**Additional file 4**

(A)

| Common name | OR gene | Potentially functional/Pseudogene | Copies | GenBank accession number |
| --- | --- | --- | --- | --- |
| Emu | EmuOR1 | Pseudogene | 1 | EU594675 |
| Emu | EmuOR2 | Functional | 1 | EU594686 |
| Emu | EmuOR3 | Functional | 1 | EU594697 |
| Emu | EmuOR4 | Functional | 1 | EU594700 |
| Emu | EmuOR5 | Functional | 3 | EU594701 |
| Emu | EmuOR6 | Functional | 4 | EU594702 |
| Emu | EmuOR7 | Functional | 5 | EU594703 |
| Emu | EmuOR8 | Functional | 1 | EU594704 |
| Emu | EmuOR9 | Pseudogene | 1 | EU594705 |
| Emu | EmuOR10 | Functional | 1 | EU594676 |
| Emu | EmuOR11 | Pseudogene | 2 | EU594677 |
| Emu | EmuOR12 | Pseudogene | 1 | EU594678 |
| Emu | EmuOR13 | Functional | 2 | EU594679 |
| Emu | EmuOR14 | Functional | 1 | EU594680 |
| Emu | EmuOR15 | Functional | 1 | EU594681 |
| Emu | EmuOR16 | Functional | 1 | EU594682 |
| Emu | EmuOR17 | Functional | 1 | EU594683 |
| Emu | EmuOR18 | Functional | 3 | EU594684 |
| Emu | EmuOR19 | Functional | 1 | EU594685 |
| Emu | EmuOR20 | Pseudogene | 2 | EU594687 |
| Emu | EmuOR21 | Functional | 1 | EU594688 |
| Emu | EmuOR22 | Functional | 1 | EU594689 |
| Emu | EmuOR23 | Functional | 1 | EU594690 |
| Emu | EmuOR24 | Functional | 1 | EU594691 |
| Emu | EmuOR25 | Functional | 1 | EU594692 |
| Emu | EmuOR26 | Functional | 1 | EU594693 |
| Emu | EmuOR27 | Functional | 1 | EU594694 |
| Emu | EmuOR28 | Pseudogene | 1 | EU594695 |
| Emu | EmuOR29 | Functional | 1 | EU594696 |
| Emu | EmuOR30 | Functional | 4 | EU594698 |
| Emu | EmuOR31 | Functional | 2 | EU594699 |
| Kiwi | KiwiOR1 | Pseudogene | 1 | EU594849 |
| Kiwi | KiwiOR2 | Functional | 1 | EU594860 |
| Kiwi | KiwiOR3 | Functional | 1 | EU594871 |
| Kiwi | KiwiOR4 | Pseudogene | 1 | EU594882 |
| Kiwi | KiwiOR5 | Functional | 1 | EU594886 |
| Kiwi | KiwiOR6 | Functional | 1 | EU594887 |
| Kiwi | KiwiOR7 | Functional | 1 | EU594888 |
| Kiwi | KiwiOR8 | Functional | 2 | EU594889 |
| Kiwi | KiwiOR9 | Functional | 1 | EU594890 |
| Kiwi | KiwiOR10 | Functional | 1 | EU594850 |
| Kiwi | KiwiOR11 | Peudogene | 1 | EU594851 |
| Kiwi | KiwiOR12 | Functional | 1 | EU594852 |
| Kiwi | KiwiOR13 | Functional | 1 | EU594853 |
| Kiwi | KiwiOR14 | Pseudogene | 1 | EU594854 |
| Kiwi | KiwiOR15 | Functional | 1 | EU594855 |
| Kiwi | KiwiOR16 | Functional | 1 | EU594856 |
| Kiwi | KiwiOR17 | Functional | 5 | EU594857 |
| Kiwi | KiwiOR18 | Functional | 1 | EU594858 |
| Kiwi | KiwiOR19 | Pseudogene | 1 | EU594859 |
| Kiwi | KiwiOR20 | Functional | 1 | EU594861 |
| Kiwi | KiwiOR21 | Functional | 1 | EU594862 |
| Kiwi | KiwiOR22 | Functional | 2 | EU594863 |
| Kiwi | KiwiOR23 | Functional | 1 | EU594864 |
| Kiwi | KiwiOR24 | Functional | 1 | EU594865 |
| Kiwi | KiwiOR25 | Pseudogene | 1 | EU594866 |
| Kiwi | KiwiOR26 | Functional | 1 | EU594867 |
| Kiwi | KiwiOR27 | Pseudogene | 1 | EU594868 |
| Kiwi | KiwiOR28 | Functional | 1 | EU594869 |
| Kiwi | KiwiOR29 | Functional | 1 | EU594870 |
| Kiwi | KiwiOR30 | Functional | 1 | EU594872 |
| Kiwi | KiwiOR31 | Functional | 1 | EU594873 |
| Kiwi | KiwiOR32 | Functional | 2 | EU594874 |
| Kiwi | KiwiOR33 | Functional | 1 | EU594875 |
| Kiwi | KiwiOR34 | Functional | 1 | EU594876 |
| Kiwi | KiwiOR35 | Functional | 1 | EU594877 |
| Kiwi | KiwiOR36 | Functional | 1 | EU594878 |
| Kiwi | KiwiOR37 | Functional | 1 | EU594879 |
| Kiwi | KiwiOR38 | Functional | 1 | EU594880 |
| Kiwi | KiwiOR39 | Functional | 1 | EU594881 |
| Kiwi | KiwiOR40 | Pseudogene | 1 | EU594883 |
| Kiwi | KiwiOR41 | Pseudogene | 1 | EU594884 |
| Kiwi | KiwiOR42 | Functional | 2 | EU594885 |
| Ostrich | OstrichOR1 | Functional | 1 | EU594796 |
| Ostrich | OstrichOR2 | Pseudogene | 5 | EU594807 |
| Ostrich | OstrichOR3 | Functional | 6 | EU594814 |
| Ostrich | OstrichOR4 | Functional | 5 | EU594815 |
| Ostrich | OstrichOR5 | Functional | 1 | EU594816 |
| Ostrich | OstrichOR6 | Functional | 1 | EU594817 |
| Ostrich | OstrichOR7 | Functional | 1 | EU594818 |
| Ostrich | OstrichOR8 | Functional | 1 | EU594819 |
| Ostrich | OstrichOR9 | Pseudogene | 2 | EU594820 |
| Ostrich | OstrichOR10 | Functional | 1 | EU594797 |
| Ostrich | OstrichOR11 | Functional | 2 | EU594798 |
| Ostrich | OstrichOR12 | Functional | 1 | EU594799 |
| Ostrich | OstrichOR13 | Functional | 3 | EU594800 |
| Ostrich | OstrichOR14 | Functional | 1 | EU594801 |
| Ostrich | OstrichOR15 | Functional | 1 | EU594802 |
| Ostrich | OstrichOR16 | Pseudogene | 1 | EU594803 |
| Ostrich | OstrichOR17 | Functional | 1 | EU594804 |
| Ostrich | OstrichOR18 | Functional | 1 | EU594805 |
| Ostrich | OstrichOR19 | Functional | 3 | EU594806 |
| Ostrich | OstrichOR20 | Functional | 3 | EU594808 |
| Ostrich | OstrichOR21 | Functional | 2 | EU594809 |
| Ostrich | OstrichOR22 | Pseudogene | 1 | EU594810 |
| Ostrich | OstrichOR23 | Functional | 1 | EU594811 |
| Ostrich | OstrichOR24 | Functional | 1 | EU594812 |
| Ostrich | OstrichOR25 | Functional | 4 | EU594813 |
| Rhea | RheaOR1 | Functional | 1 | EU594821 |
| Rhea | RheaOR2 | Functional | 3 | EU594832 |
| Rhea | RheaOR3 | Functional | 6 | EU594842 |
| Rhea | RheaOR4 | Functional | 5 | EU594843 |
| Rhea | RheaOR5 | Pseudogene | 2 | EU594844 |
| Rhea | RheaOR6 | Functional | 2 | EU594845 |
| Rhea | RheaOR7 | Pseudogene | 1 | EU594846 |
| Rhea | RheaOR8 | Functional | 1 | EU594847 |
| Rhea | RheaOR9 | Functional | 1 | EU594848 |
| Rhea | RheaOR10 | Functional | 1 | EU594822 |
| Rhea | RheaOR11 | Functional | 1 | EU594823 |
| Rhea | RheaOR12 | Functional | 2 | EU594824 |
| Rhea | RheaOR13 | Functional | 1 | EU594825 |
| Rhea | RheaOR14 | Pseudogene | 1 | EU594826 |
| Rhea | RheaOR15 | Functional | 1 | EU594827 |
| Rhea | RheaOR16 | Functional | 1 | EU594828 |
| Rhea | RheaOR17 | Pseudogene | 3 | EU594829 |
| Rhea | RheaOR18 | Pseudogene | 2 | EU594830 |
| Rhea | RheaOR19 | Pseudogene | 1 | EU594831 |
| Rhea | RheaOR20 | Functional | 1 | EU594833 |
| Rhea | RheaOR21 | Pseudogene | 4 | EU594834 |
| Rhea | RheaOR22 | Functional | 1 | EU594835 |
| Rhea | RheaOR23 | Functional | 1 | EU594836 |
| Rhea | RheaOR24 | Functional | 2 | EU594837 |
| Rhea | RheaOR25 | Functional | 1 | EU594838 |
| Rhea | RheaOR26 | Functional | 1 | EU594839 |
| Rhea | RheaOR27 | Functional | 1 | EU594840 |
| Rhea | RheaOR28 | Functional | 2 | EU594841 |

(B)

| Common name | OR gene | Potentially functional/Pseudogene | Copies | GenBank accession number |
| --- | --- | --- | --- | --- |
| Kaka | KakaOR1 | Functional | 1 | EU594706 |
| Kaka | KakaOR2 | Functional | 1 | EU594717 |
| Kaka | KakaOR3 | Functional | 3 | EU594721 |
| Kaka | KakaOR4 | Functional | 1 | EU594722 |
| Kaka | KakaOR5 | Functional | 2 | EU594723 |
| Kaka | KakaOR6 | Functional | 2 | EU594724 |
| Kaka | KakaOR7 | Functional | 1 | EU594725 |
| Kaka | KakaOR8 | Functional | 2 | EU594726 |
| Kaka | KakaOR9 | Functional | 2 | EU594727 |
| Kaka | KakaOR10 | Pseudogene | 1 | EU594707 |
| Kaka | KakaOR11 | Functional | 1 | EU594708 |
| Kaka | KakaOR12 | Functional | 2 | EU594709 |
| Kaka | KakaOR13 | Functional | 11 | EU594710 |
| Kaka | KakaOR14 | Functional | 1 | EU594711 |
| Kaka | KakaOR15 | Pseudogene | 2 | EU594712 |
| Kaka | KakaOR16 | Functional | 1 | EU594713 |
| Kaka | KakaOR17 | Pseudogene | 1 | EU594714 |
| Kaka | KakaOR18 | Functional | 2 | EU594715 |
| Kaka | KakaOR19 | Functional | 3 | EU594716 |
| Kaka | KakaOR20 | Pseudogene | 5 | EU594718 |
| Kaka | KakaOR21 | Pseudogene | 1 | EU594719 |
| Kaka | KakaOR22 | Functional | 1 | EU594720 |
| Kakapo | KakapoOR1 | Functional | 1 | EU594728 |
| Kakapo | KakapoOR2 | Functional | 1 | EU594739 |
| Kakapo | KakapoOR3 | Functional | 1 | EU594750 |
| Kakapo | KakapoOR4 | Functional | 1 | EU594760 |
| Kakapo | KakapoOR5 | Functional | 1 | EU594761 |
| Kakapo | KakapoOR6 | Functional | 1 | EU594762 |
| Kakapo | KakapoOR7 | Functional | 3 | EU594763 |
| Kakapo | KakapoOR8 | Functional | 2 | EU594764 |
| Kakapo | KakapoOR9 | Functional | 5 | EU594765 |
| Kakapo | KakapoOR10 | Functional | 1 | EU594729 |
| Kakapo | KakapoOR11 | Functional | 1 | EU594730 |
| Kakapo | KakapoOR12 | Functional | 4 | EU594731 |
| Kakapo | KakapoOR13 | Functional | 1 | EU594732 |
| Kakapo | KakapoOR14 | Functional | 1 | EU594733 |
| Kakapo | KakapoOR15 | Functional | 1 | EU594734 |
| Kakapo | KakapoOR16 | Pseudogene | 1 | EU594735 |
| Kakapo | KakapoOR17 | Functional | 1 | EU594736 |
| Kakapo | KakapoOR18 | Pseudogene | 1 | EU594737 |
| Kakapo | KakapoOR19 | Functional | 1 | EU594738 |
| Kakapo | KakapoOR20 | Pseudogene | 1 | EU594740 |
| Kakapo | KakapoOR21 | Pseudogene | 1 | EU594741 |
| Kakapo | KakapoOR22 | Pseudogene | 1 | EU594742 |
| Kakapo | KakapoOR23 | Functional | 1 | EU594743 |
| Kakapo | KakapoOR24 | Pseudogene | 1 | EU594744 |
| Kakapo | KakapoOR25 | Pseudogene | 1 | EU594745 |
| Kakapo | KakapoOR26 | Functional | 1 | EU594746 |
| Kakapo | KakapoOR27 | Pseudogene | 1 | EU594747 |
| Kakapo | KakapoOR28 | Pseudogene | 1 | EU594748 |
| Kakapo | KakapoOR29 | Functional | 1 | EU594749 |
| Kakapo | KakapoOR30 | Pseudogene | 1 | EU594751 |
| Kakapo | KakapoOR31 | Functional | 1 | EU594752 |
| Kakapo | KakapoOR32 | Functional | 1 | EU594753 |
| Kakapo | KakapoOR33 | Functional | 1 | EU594754 |
| Kakapo | KakapoOR34 | Functional | 2 | EU594755 |
| Kakapo | KakapoOR35 | Functional | 2 | EU594756 |
| Kakapo | KakapoOR36 | Pseudogene | 1 | EU594757 |
| Kakapo | KakapoOR37 | Functional | 1 | EU594758 |
| Kakapo | KakapoOR38 | Pseudogene | 1 | EU594759 |
| Kea | KeaOR1 | Functional | 1 | EU594766 |
| Kea | KeaOR2 | Functional | 1 | EU594777 |
| Kea | KeaOR3 | Pseudogene | 1 | EU594788 |
| Kea | KeaOR4 | Functional | 2 | EU594790 |
| Kea | KeaOR4 | Functional | 4 | EU594791 |
| Kea | KeaOR6 | Functional | 1 | EU594792 |
| Kea | KeaOR7 | Functional | 1 | EU594793 |
| Kea | KeaOR8 | Functional | 5 | EU594794 |
| Kea | KeaOR9 | Functional | 1 | EU594795 |
| Kea | KeaOR10 | Functional | 4 | EU594767 |
| Kea | KeaOR11 | Functional | 1 | EU594768 |
| Kea | KeaOR12 | Functional | 1 | EU594769 |
| Kea | KeaOR13 | Functional | 1 | EU594770 |
| Kea | KeaOR14 | Functional | 1 | EU594771 |
| Kea | KeaOR15 | Functional | 1 | EU594772 |
| Kea | KeaOR16 | Functional | 1 | EU594773 |
| Kea | KeaOR17 | Functional | 1 | EU594774 |
| Kea | KeaOR18 | Functional | 1 | EU594775 |
| Kea | KeaOR19 | Functional | 2 | EU594776 |
| Kea | KeaOR20 | Pseudogene | 3 | EU594778 |
| Kea | KeaOR21 | Functional | 1 | EU594779 |
| Kea | KeaOR22 | Functional | 2 | EU594780 |
| Kea | KeaOR23 | Functional | 1 | EU594781 |
| Kea | KeaOR24 | Functional | 1 | EU594782 |
| Kea | KeaOR25 | Functional | 2 | EU594783 |
| Kea | KeaOR26 | Functional | 1 | EU594784 |
| Kea | KeaOR27 | Functional | 1 | EU594785 |
| Kea | KeaOR28 | Functional | 1 | EU594786 |
| Kea | KeaOR29 | Functional | 5 | EU594787 |
| Kea | KeaOR30 | Functional | 1 | EU594789 |
